# Hand Function is Altered in Individuals with a History of Illicit Stimulant Use

**DOI:** 10.1371/journal.pone.0115771

**Published:** 2014-12-29

**Authors:** Verity Pearson-Dennett, Stanley C. Flavel, Robert A. Wilcox, Dominic Thewlis, Adam P. Vogel, Jason M. White, Gabrielle Todd

**Affiliations:** 1 School of Pharmacy and Medical Sciences and Sansom Institute, University of South Australia, South Australia, Australia; 2 Department of Neurology, Flinders Medical Centre, South Australia, Australia; 3 Human Physiology, Medical School, Flinders University, South Australia, Australia; 4 School of Health Sciences and Sansom Institute, University of South Australia, South Australia, Australia; 5 Speech Neuroscience Unit, University of Melbourne, Victoria, Australia; Chiba University Center for Forensic Mental Health, Japan

## Abstract

Use of illicit stimulant drugs such as methamphetamine, cocaine, and ecstasy are a significant worldwide problem. However, little is known about the effect of these drugs on movement. The aim of the current study was to investigate hand function in adults with a history of illicit stimulant use. We hypothesized that prior use of illicit stimulant drugs is associated with abnormal manipulation of objects. The study involved 22 subjects with a history of illicit stimulant use (aged 29±8 yrs; time since last use: 1.8±4.0 yrs) and two control groups comprising 27 non-drug users (aged 25±8 yrs) and 17 cannabis users with no history of stimulant use (aged 22±5 yrs). Each subject completed screening tests (neuropsychological assessment, medical history questionnaire, lifetime drug history questionnaire, and urine drug screen) prior to gripping and lifting a light-weight object with the dominant right hand. Horizontal grip force, vertical lift force, acceleration, and first dorsal interosseus electromyographic (EMG) activity were recorded during three trials. In trial one, peak grip force was significantly greater in the stimulant group (12.8±3.9 N) than in the control groups (non-drug: 10.3±4.6 N; cannabis: 9.4±2.9 N, P<0.022). However, peak grip force did not differ between groups in trials two and three. The results suggest that individuals with a history of stimulant use overestimate the grip force required to manipulate a novel object but, are able to adapt grip force in subsequent lifts. The results suggest that movement dysfunction may be an unrecognized consequence of illicit stimulant use.

## Introduction

Illicit use of stimulant drugs is a significant worldwide problem. The most commonly used illicit stimulant drugs are amphetamine, methamphetamine (termed ‘amphetamines’), and ecstasy. 14–53 million people worldwide reported use of these drugs within the previous year [1]. The Oceania region, comprising people primarily from Australia and New Zealand, has the highest annual prevalence of illicit stimulant use in the world [1] with ∼10% of people aged 20–29 yrs reporting use of an illicit stimulant drug within the last 12 months [2].

Given the high prevalence of illicit drug use in the community, it is surprising that very little is known about the long-term effect of illicit drug use on movement. Long-lasting changes in movement are likely given that abnormalities in brain regions that control movement have been observed in humans with a history of illicit stimulant use [3], [4]. Furthermore, individuals with a history of stimulant use exhibit abnormally large tremor during finger movement [5] and poor performance on tests of hand dexterity (e.g. pegboard test) [6], [7].

The aim of the current study was to further explore hand function in individuals with a history of illicit stimulant use. We investigated hand function using a sensitive, objective, and well validated object grip and lift task. Lifting an object requires a grip force that is appropriate to overcome gravity and prevent slipping. This force is predetermined by an internal model based on the physical characteristics of the object, prior experience, sensory feedback, and the individual’s safety margin to prevent slipping [8], [9]. Pathological (e.g. stroke) [10] and physiological states (e.g. fatiguing exercise) [11] have been shown to affect properties of the grip and lift task. We hypothesized that individuals with a history of illicit stimulant use would exhibit increased grip force (i.e. higher safety margin for error) and abnormal rate of force application during object manipulation. The hypothesis is based on several observations. First, methamphetamine and ecstasy are toxic to primarily dopaminergic and serotonergic neurons, respectively [12], [13] and these monoamine neurotransmitter systems are important for control of movement. Second, case studies of clinically diagnosed movement disorders have been observed in stimulant users [14]. Third, epidemiological data suggests that methamphetamine use is associated with increased risk of developing Parkinson’s disease later in life (2.65 hazard ratio) [15] and increased grip force and decreased rate of grip force application have been observed in *de novo* Parkinson’s disease patients [16]. The results of the current study will further our understanding of a potentially under recognized consequence of illicit stimulant use.

## Materials and Methods

### Ethics Statement

All experimental procedures were approved by the Human Research Ethics Committee at the University of South Australia. Experimental procedures were conducted according to The Code of Ethics of the World Medical Association (Declaration of Helsinki). Written informed consent was obtained prior to participation.

### Subjects and screening

Hand function was assessed in 66 adults aged 18–48 yrs (see Table 1). Three groups of subjects were investigated: 22 subjects with a history of illicit stimulant use and two control groups comprising 27 non-drug users and 17 cannabis users (with no history of stimulant use). All subjects were recruited from the community and only right-hand dominant subjects were included in the study. The inclusion criteria for the stimulant group were use of amphetamine, methamphetamine, ecstasy, and/or cocaine on ≥7 occasions. Inclusion criteria for the cannabis control group were cannabis use on ≥7 occasions but no history of illicit stimulant use. Inclusion criteria for the non-drug control group was cannabis use on ≤2 occasions and no other history of illicit drug use (alcohol and tobacco use was permitted). The study was conducted in Adelaide, Australia.

**Table 1:** Subject characteristics for the control, stimulant, and cannabis groups.

Characteristic	Control (n = 27)	Stimulant (n = 22)	Cannabis (n = 17)
Age (yrs)	25±8	29±8* ^,^ †	22±5
Sex	16 M, 11 F	13 M, 9 F	11 M, 6 F
Education (yrs)	15±2	15±3	16±2
BDI–II score	4±5	9±7*	8±9
Drug overdose	0	4	0
Lifetime alcohol (total drinks)	1,283±4,403	6,609±7,227* ^,^ †	2,146±3,072*
Lifetime tobacco (total cigarettes)	8±9	32,018±50,630* ^,^ †	2,145±6,727*

Data are mean ± SD.

^*^Significantly different from control group (P<0.05).

† Significantly different from cannabis group (P<0.05).

Subjects underwent a series of screening tests prior to participation. Subjects were asked to complete a brief medical history questionnaire and provide a urine sample for routine drug screening (PSCupA-6MBAU, US Diagnostics Inc., Huntsville, Alabama, USA). Urine data is missing for one control subject due to mislabelling. All subjects were then asked to complete an in-house drug history questionnaire to document lifetime and recent use of alcohol, tobacco, and illicit drugs. The questionnaire listed 20 illicit drugs and requested information on other illicit drugs not listed. Items on the questionnaire included age of first use, age of regular use, duration of use, frequency of use (current and lifetime), number of times used in the last year, average dose (current and lifetime), frequency of high dose use, and time since last use defined for each drug. The number of drug overdoses and any treatment for drug dependency were also noted. The final screening test involved a neuropsychological assessment to ensure that all participants had normal memory and cognition. Four cognitive domains were assessed with the following tests: Logical Memory I and II [17], Verbal Trails [18], Verbal Fluency [18], [19], and Digit Span forwards and backwards [20]. Performance on each test was compared to published normative data matched for age and years of education. Symptoms of depression (over the past two weeks) were also assessed with a 21 item self-report rating scale (Beck Depression Inventory) [21].

Exclusion criteria included: a) history of neurological damage and/or neurological illness, b) non-neurological conditions that may affect movement (e.g. arthritis in the hand or carpel tunnel syndrome), c) current use of medications that may affect movement (e.g. antidepressants, benzodiazepines, beta blockers), d) frequent illicit opioid use (i.e. >5 times), and e) positive urine drug test for amphetamine, methamphetamine, MDMA (3,4-methylenedioxymethamphetamine or ‘ecstasy’), cocaine, opioids, and/or benzodiazepines. Subjects who tested positive for cannabis were allowed to participate if use was greater than 12**hrs prior to the experiment. This exemption was due to the metabolite of the main active ingredient of cannabis (tetrahydrocannabinol) remaining in body fat for up to 90 days after last use. Subjects were also excluded if poor performance was observed on two or more of the cognitive domains tested. Poor performance was defined as greater than two standard deviations below the mean of published normative data for Digit Span [22], Verbal Fluency [23], and Logical Memory I and II [24] and performance greater than two standard deviations above the mean for Verbal Trails [25].

### Experimental protocol

**Figure 1:**
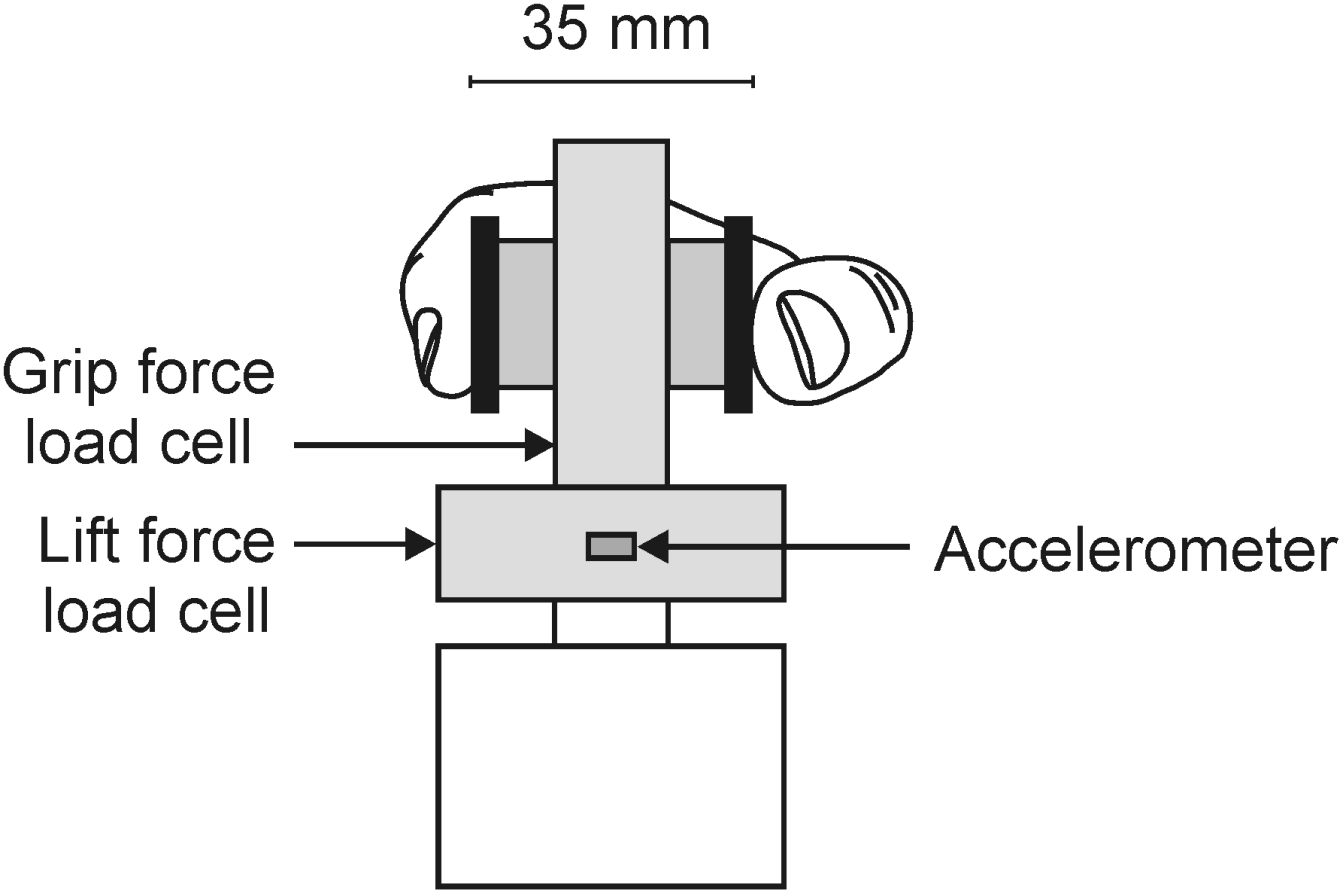
Experimental apparatus used in task one (total weight 0.342 kg). The index finger and thumb contacted the test object on two polished brass disks positioned 35**mm apart.

Subjects sat on a chair in front of a table and completed a modified version of the Edinburgh Handedness Inventory [26] to confirm right hand dominance. Subjects then performed three tasks with the right hand. The first task involved gripping and lifting a light-weight object (342**g; see Fig. 1). The test object consisted of two load cells (model MPL-100; Transducer Techniques, Temecula, CA, USA) mounted orthogonally for measurement of horizontal grip force and vertical lift force. The grip load cell was mounted between two polished brass disks, 35**mm apart. A dual axis accelerometer (±2**g, model ADXL311J, RS Components Pty Ltd, Smithfield, Australia) was also attached to the apparatus. Subjects were instructed to ‘Lift the object off the table to the height indicated (∼10**cm). Hold the object there for 3**s and then replace it on the table’. The task (duration ∼6**s) was performed with the index finger and thumb (pinch grip) and the lifting movement occurred primarily through elbow flexion. The experimenter demonstrated the task prior to the subjects’ first attempt and no practice was allowed. Subjects performed three trials, with trials commencing at ∼10**s intervals. Three trials is sufficient for adaptation of grip force to a novel object [27].

The second task involved three brief maximal voluntary contractions (MVCs; 2–3**s duration) to enable normalization of some parameters measured during the grip and lift task. MVCs were performed with a pinch grip and subjects were not required to lift the object off the table. MVCs were separated by ∼1**min to minimize fatigue. Subjects were given verbal encouragement and visual feedback of force production during MVCs.

The third task involved testing hand dexterity with the grooved pegboard test (for comparison to previous literature). Subjects were instructed to place key-shaped pegs into corresponding holes (model 32025, Lafayette Instrument Company, Lafayette IN, USA), in a set sequence and as fast as possible. Subjects performed three trials with 1**min rest between. The time to complete each trial was recorded.

### Data recordings

During tasks one and two, electromyographic activity (EMG) was recorded with surface electrodes (Ag–AgCl, 10**mm in diameter) over the muscle belly (active) and tendon (reference) of the first dorsal interosseus muscle (FDI; inter-electrode distance: 3**cm). Data were sampled at 2000**Hz with a data acquisition system (CED 1902 Amplifier and Power 1401 Interface) and specialized software (Spike2; Cambridge Electronic Design, Cambridge, UK). Surface EMG was amplified (x300) and band-pass filtered (2nd order Butterworth filter, 20–1000 Hz). Force and acceleration signals were sampled at 400**Hz using the same data acquisition system. The signals were amplified (force: x1000; acceleration: x3) and low-pass filtered (2nd order Butterworth filter, 100**Hz).

### Data Analysis

The grip and lift task (task one) was divided into two phases: lift (dynamic) phase and hold (stationary) phase. The lift phase was defined as the period between 0**s (lift onset) and 1.5**s and the hold phase was defined as the period between 1.5**s and 2.5**s. A total of 15 parameters were measured in the lift phase and three parameters were measured in the hold phase. The variety of parameters provided information about grip strategy, kinetics and kinematics of the lifting movement, and coupling between grip force and lift force. In the lift phase, grip and lift onset were determined by first applying a 20**Hz low-pass filter to the raw data followed by calculation of the rate of change in grip force (dGF/dt) and lift force (dLF/dt). Onset was defined as the time of the initial rise in the force derivative (above the noise level) that initiated the rise to the maximum derivative. This procedure was used because the change in lift force represents the first measurable mechanical event in lifting the object. Grip onset relative to lift onset (termed ‘preload duration’) and maximum rate of change in force (dGF/dtmax and dLF/dtmax) were also measured. The temporal relationship between grip force and lift force was assessed by cross-correlation of the rate of change in grip force (dGF/dt) and lift force (dLF/dt) [28]. dLF/dt was shifted in increments of 2.5**ms (sampling resolution) relative to dGF/dt until the maximum cross-correlation coefficient (ρ) was obtained (IBM SPSS Statistics 20, Armonk NY, USA). The time shift required to achieve the maximum cross-correlation coefficient represents the time difference between the change in lift force and the change in grip force, and is an index of whether the grip strategy was primarily anticipatory (i.e. grip force leads lift force, negative time shift) or reactive (i.e. grip force lags lift force, positive time shift). Other parameters that were measured in the lift phase include peak force and acceleration, time-to-peak force and acceleration, minimum lift force (degree of downward push before lifting the object), and root mean square (RMS) EMG, all of which were measured from raw traces. In the hold phase, mean grip force, grip force coefficient of variation (%), and RMS EMG were measured from raw traces. For brief MVCs, mean grip force and RMS EMG was measured over a 1**s period.

### Statistical analysis

Group data are presented as means ± standard deviation in the text and mean ± standard error of the mean in figures. One-way ANOVA was used to compare subject characteristics, MVC force, alcohol and tobacco use between groups. Non-parametric data were transformed to ranks and one-way ANOVA on ranks were performed. Post hoc discrimination was made with a Bonferroni procedure. Independent Student’s t-test was used for between-group comparison (stimulant versus cannabis) of cannabis use and the score on the agitation and irritability questions of the BDI–II. For task one and three, group data were analysed with two-way repeated measures ANOVA with gender as a covariate for comparison of group (control, stimulant, cannabis; between-subject factor) and trial (one, two, three; within-subject factor). Mauchly’s test of sphericity was performed and the Greenhouse–Geisser method was used to correct for non-sphericity (IBM SPSS Statistics 20, Armonk NY, USA). Post hoc discrimination was made with a sequential Bonferroni procedure. Spearman Rank Order correlation was used to investigate the relationship between peak grip force and drug-use characteristics (trial one only) in the stimulant group. The relation between peak grip force and maximum rate of grip force application (dGF/dtmax) was investigated with linear regression analysis (SigmaPlot 11.0; Systat Software Inc, San Jose CA, USA). Statistical significance was set at P<0.05.

## Results

### Subject characteristics

Table 1 shows the characteristics of each group. There was a significant main effect of group on age (F_2,62_ = 6.982, P = 0.002), symptoms of depression (F_2,62_ = 4.681, P = 0.013), and lifetime alcohol (F_2,62_ = 23.483, P<0.001) and tobacco use (F_2,62_ = 16.142, P<0.001). The age of subjects in the stimulant group (29±8 yrs) was higher than subjects in the non-drug control group (25±8 yrs; P = 0.032) and cannabis group (22±5 yrs; P = 0.002) but the groups were well matched for years of education. Subjects in the stimulant group (P = 0.014) reported more symptoms of depression (BDI–II score) than subjects in the non-drug control group but symptoms of depression did not differ between the drug using groups. The score on the agitation (0.55±0.60 vs 0.59±0.71) and irritability (0.64±0.66 vs 0.29±0.77) questions of the BDI–II also did not differ between the stimulant and cannabis groups. All subjects exhibited normal neuropsychological performance and performance on the neuropsychological tests did not significantly differ between groups. As expected, use of alcohol and tobacco differed between groups (P<0.001). Lifetime use of alcohol and tobacco was greatest in the stimulant group and least in the non-drug control group (P<0.001).

Table 2 shows history of illicit drug use in the stimulant and cannabis groups. In the stimulant group, ecstasy was the most commonly used stimulant but, use of methamphetamine was more frequent (i.e. number of occasions). Duration of abstinence from any stimulant drug ranged from 3 days to 15 yrs (mean: 1.9±4.0 yrs, median: 1.5 months) and the average duration of abstinence from ecstasy and amphetamines was 2.4±4.0 yrs (median: 8 months) and 5.6±7.8 yrs (median: 1.6 yrs), respectively. As expected, use of other classes of drugs (poly-drug use) was common. All subjects in the stimulant group reported prior use of cannabis and the average duration of abstinence from cannabis did not differ between the stimulant (mean: 73±102 days; median: 45 days) and cannabis (mean: 315±500 days; median: 30 days) groups. A large percentage of subjects in the stimulant group reported prior use of hallucinogens (primarily lysergic acid diethylamide or ‘LSD’) but use of hallucinogens was less common in the cannabis group. Illicit use of sedatives and opiates was minimal. Five subjects in total (three in the stimulant group) had a positive urine screen for cannabis, but none of the subjects reported cannabis use in the 12**hrs prior to testing.

**Table 2:** Illicit drug use in the stimulant and cannabis groups.

Drug Class	Stimulant (n = 22)	Cannabis (n = 17)
Stimulants	100% (206±334)	0%
Ecstasy	96% (75±121)	0%
Methamphetamine	68% (192±316)	0%
Cocaine	55% (6±8)	0%
Pharmaceutical	9% (4±3)	0%
Cannabis	100% (2,246±2,770)	100% (123±118)
Hallucinogens	82% (60±154)	24% (5±4)
Inhalants	50% (52±78)	12% (162±213)
Sedatives	31% (6±9)	6% (8)
Opiates	36% (8±15)	0%

Data are percentage of subjects that have consumed that class of illicit drug in their lifetime and mean ± SD for number of occasions used (in brackets). The term ‘hallucinogen’ describes LSD (lysergic acid diethylamide), LSA (d-lysergic acid amide), ‘magic’ mushrooms, DOI (2,5-dimethoxy-4-iodoamphetamine), salvia divinorum, and/or ketamine. The term ‘opiate’ describes heroin, opium, and recreational use of codeine, oxycodone, methadone, and/or morphine. The term ‘inhalant’ describes amyl nitrate, nitrous oxide, and ethyl chloride. The term ‘sedative’ describes GHB/fantasy and recreational use of benzodiazepine, antidepressants, and antihistamines

### Object grip and lift

**Figure 2:**
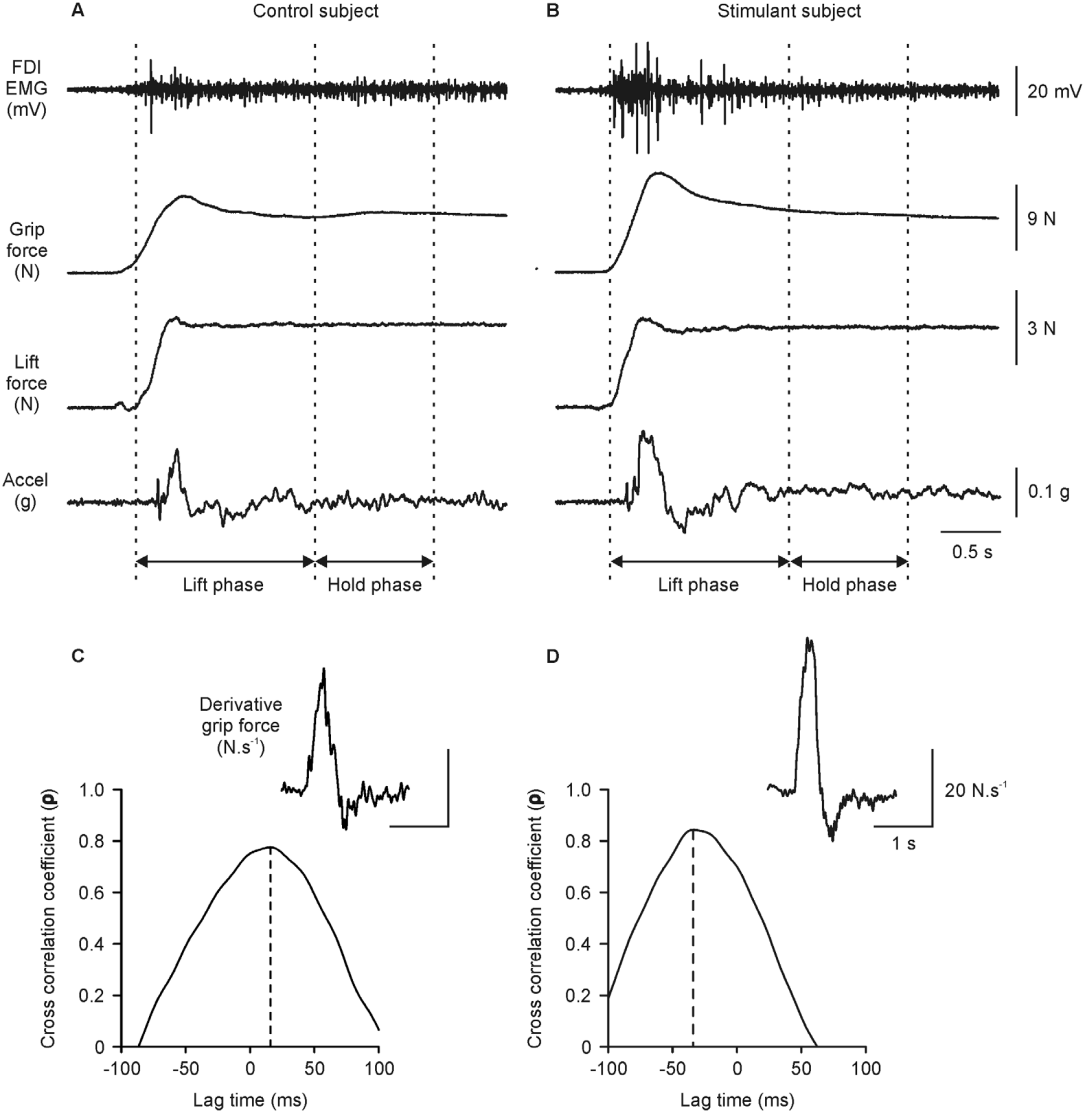
Raw data from one control subject (left panels) and one stimulant subject (right panels) during trial one of the grip and lift task. A and B) Raw traces of first dorsal interosseus EMG (top panel), grip force (2^nd^ panel), lift force (3^rd^ panel), and acceleration (bottom panel). The task was divided into two phases: lift (dynamic) phase and hold (stationary) phase. The lift phase ranged from 0**s (lift onset) to 1.5**s and the hold phase ranged from 1.5 to 2.5**s. C and D) Temporal characteristics of grip force and lift force. The derivative of grip force (i.e. dGF/dt, inset) was correlated with the derivative of lift force (dLF/dt) and the resultant cross-correlogram is shown. Vertical dashed lines in C and D represent the time shift required to achieve the maximal cross-correlation coefficient.

Examples of raw data from one control subject and one stimulant subject are shown in Fig. 2A and B, respectively. Grip force and lift force increased in parallel before the object moved. After the object began to move (increase in acceleration), grip force and lift force continued to increase until reaching a peak. Grip force then decreased slightly and plateaued while the object was held stationary. The magnitude of the plateau in grip force was ∼2.5 times that of lift force, indicating a large safety margin to prevent slipping. Examples of the temporal characteristics of grip force and lift force are shown in Figs. 2C and D. Data in Fig. 2 suggest that the stimulant subject performed the task with a higher grip force and rate of grip force application than the control subject.

**Figure 3:**
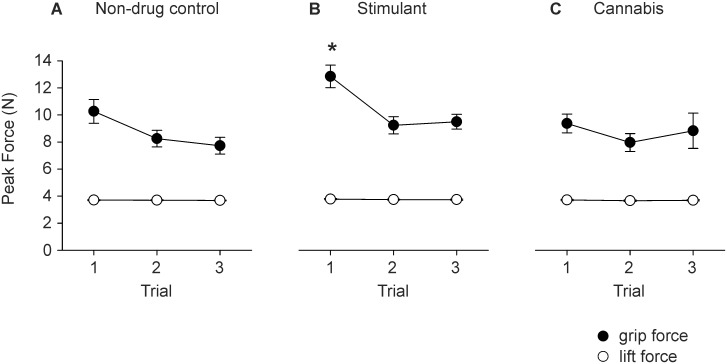
Group data (mean ± sem) for peak force measured in the lift phase. Peak grip force (black symbols) and lift force (white symbols) are shown for trials one to three. A) Non-drug control group. B) Stimulant group. C) Cannabis group. * Significantly different from non-drug control group (P = 0.022) and cannabis group (P = 0.013).

Fig. 3 shows group data for peak grip force during the lift phase. There was a significant main effect of trial on peak grip force (raw: F_2,124_ = 8.822, P = 0.001; normalised: F_2,124_ = 3.968, P = 0.021). Peak grip force decreased from trial one to trials two and three (P<0.025). Peak grip force did not significantly differ between groups but there was a significant group-by-trial interaction. The interaction reached statistical significance for raw peak grip force (F_4,124_ **= **2.574, P = 0.049) but not normalised peak grip force (%MVC; F_4,124_ = 2.338, P = 0.059). In trial one, peak grip force was significantly greater in the stimulant group than in the two control groups (P<0.022) but, peak grip force did not differ between the two control groups. In the stimulant group, there was no significant correlation between peak grip force and a) lifetime stimulant use (number of occasions), b) duration of abstinence from stimulant use, c) lifetime alcohol use, d) lifetime tobacco use, and e) lifetime cannabis use.

Fig. 3 also shows group data for peak lift force. Peak lift force did not differ between trials or groups but, there was a significant main effect of group on minimum lift force (F_2,62_ = 4.347, P = 0.017). Minimum lift force was significantly larger in the stimulant group (−0.25±0.17 N) than in the non-drug control group (−0.21±0.18 N, P = 0.016) and cannabis group (−0.20±0.22 N, P = 0.025), but minimum lift force did not differ between the two control groups.

In the hold phase, mean grip and lift force did not differ between groups. Mean grip force also did not differ between trials but, a significant main effect of trial was observed for mean lift force (F_2,124_ = 11.371, P<0.001). Mean lift force tended to be lower in the stimulant group (3.34±0.06 N) and cannabis group (3.34±0.07 N) than in the non-drug control group (3.35±0.05 N) but post hoc analysis did not reach statistical significance (data not shown).

**Figure 4:**
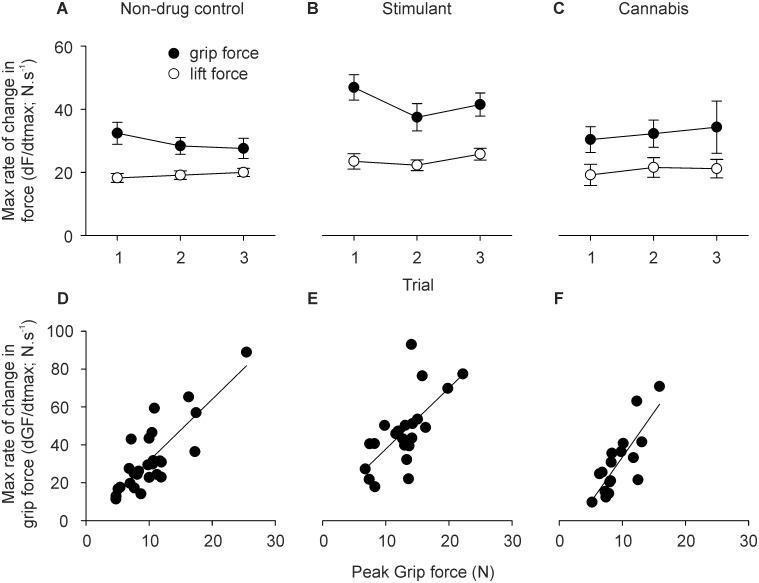
Maximum rate of change in force (i.e. maximum derivative of force) during the lift phase. A–C) Group data (mean ± sem) for grip force (black symbols) and lift force (white symbols) in trials one to three for the A) non-drug control group, B) stimulant group, and C) cannabis group. D–F) Single-subject data showing the relationship between peak grip force and maximum rate of change in grip force in the D) non-drug control group, E) stimulant group, and F) cannabis group. Solid line shows result of linear regression analysis (P<0.001).

Fig. 4A–C shows group data for the maximum rate of change in force during the lift phase. There was a significant main effect of group but not trial on the maximum rate of change in grip force (dGF/dtmax: F_2,62_ = 3.516, P = 0.036) and lift force (dLF/dtmax: F_2,62_ = 3.892, P = 0.026). The maximum rate of change in grip force and lift force was significantly greater in the stimulant group than the non-drug control group (P = 0.016), but did not differ between the two control groups. A significant linear relation was observed between peak grip force and maximum rate of change in grip force for each group (assessed in trial one; P<0.001; Fig. 4D–F).

**Figure 5:**
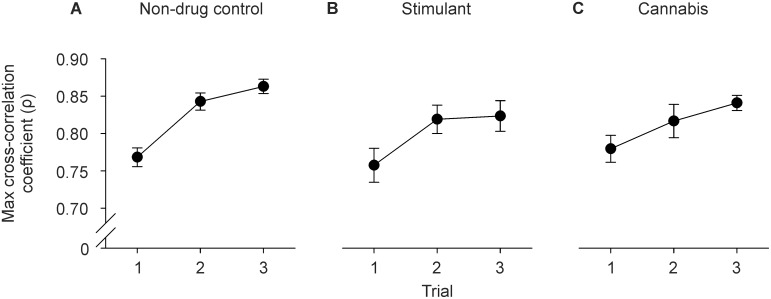
Group data (mean ± sem) showing the maximum cross-correlation coefficient in the A) non-drug control group, B) stimulant group, and C) cannabis group.

Fig. 5 shows group data for the temporal relation between grip and lift force (i.e. maximum cross-correlation coefficient). There was a significant main effect of trial on the maximum cross-correlation coefficient (F_2,124_ = 7.188, P<0.001). The maximum cross-correlation coefficient increased between trial one and trials two-three (P<0.025). However, the maximum cross-correlation coefficient did not differ between groups.

No other parameters differed between groups or trials.

### Maximal voluntary pinch contraction

Force during the maximal voluntary pinch contraction differed between groups (F_2,62_ **= **5.594, P = 0.006). Maximal force was significantly lower in the non-drug control group (53.3±15.4 N) than in the stimulant (64.7±20.3 N; P = 0.026) and cannabis (65.8±17.9 N; P = 0.015) groups. However, maximal force did not differ between the two drug groups.

### Grooved pegboard

There was a significant main effect of trial on the grooved pegboard test (F_2,124_ = 14.511, P<0.001). Performance time decreased between trial one (64.7±11.2**s) and trials two (57.6±8.9**s; P = 0.017) and three (55.1±8.3**s; P = 0.025). However, performance did not differ between groups.

## Discussion

Object manipulation tasks were used to investigate hand function in adults with a history of illicit stimulant use. The results of our study show, for the first time, that individuals with a history of illicit stimulant use employ a different strategy to manipulate novel objects than non-stimulant users.

When lifting a novel object (i.e. trial one), individuals with a history of illicit stimulant use generate a significantly larger grip force than non-stimulant users. The grip force measured in individuals with a history of stimulant use was 25% and 37% larger than that measured in non-drug users and cannabis users, respectively. This suggests that individuals with a history of stimulant use overestimate the amount of grip force required to lift a novel object and consequently, manipulate new objects in a less efficient manner and with a higher safety margin for error. The higher rate of grip force application observed in stimulant users could also conceivably lead to inaccuracy of fine movements, but this was not evident during the grooved pegboard test.

Previous research suggests that estimation of grip force is determined largely prior to contact with a novel object. It is thought to involve an internal model that is based on the physical properties of the object, prior experience, sensory feedback, and the individual’s safety margin to prevent slip [9], [8]. Our results suggest that this internal model may be altered in individuals with a history of illicit stimulant use.

The overestimation of grip force during manipulation of novel objects was observed in a population of stimulant users that had largely not used stimulant drugs for several months (mean: 1.9±4.0 yrs, median: 1.5 months). This suggests that overestimation of grip force may be a long-lasting consequence of illicit stimulant use. It is unclear if recovery occurs over time but the lack of a correlation between grip force and duration of abstinence is a worrying finding.

The overestimation of grip force required to lift a novel object in individuals with a history of illicit stimulant use was not related to strength of the muscles involved in the task. Maximal strength in the stimulant group was comparable to that of the cannabis group, yet cannabis users did not exhibit augmented grip force during manipulation of a novel object. It is unclear why a significant difference in maximal pinch grip was observed between the drug using and non-drug using groups. There does not appear to be an acute effect of amphetamine or cannabis use on maximal grip strength in healthy adults [29], [30] and there is no prior evidence for long lasting effects of these drugs on maximal grip force. Future studies will investigate this finding further.

Between group differences in the grip force used to lift a novel object was observed in trial one but not in trials two and three. This suggests that individuals with a history of illicit stimulant use are able to correct their mistake and perform the task in a similar manner to control subjects in subsequent trials. Thus, grip force adaptation and learning of this task appear to be preserved in individuals with a history of stimulant use. This may explain why performance on the grooved pegboard test, involving 25 consecutive peg placements, was unaltered in the stimulant group. Normal performance on the grooved pegboard test also suggests that movement speed, and possibly sensorimotor processing, are unaffected in individuals with a history of stimulant use.

The underling mechanism responsible for over-estimation of grip force during manipulation of novel objects in the stimulant group is difficult to identify. The effect is unlikely to be associated with cannabis use, neuropsychological factors, age, and peak lift force. Evidence that support this view is inclusion of a cannabis control group comprising individuals with a history of cannabis use but no history of illicit stimulant use. Over-estimation of grip force was not observed in the cannabis group and peak grip force was significantly larger in the stimulant group than in the cannabis group during trial one. Second, all subjects in the current study exhibited normal performance on tests of memory and cognition and, neuropsychological performance did not differ between groups. Furthermore, symptoms of depression (quantified with the BDI–II) did not differ between the stimulant and cannabis groups. Acute nervousness and anxiety were not measured in the current study but are unlikely to have differed between the stimulant and cannabis groups given that the response to the agitation and irritability questions on the BDI–II did not significantly differ between the drug using groups. Third, the age of subjects in the stimulant group (29±8 yrs) was higher than subjects in the non-drug control group (25±8 yrs) and cannabis group **(**22±5 yrs) but, peak grip force in the grip and lift task does not differ between healthy adults aged 18–30 yrs and 50–65 yrs [31]. However, one can only speculate about other contributing factors due to methodological limitations associated with all studies on illicit drug use in humans. The two most important limitations are poly-drug use (i.e. use of more than one psychoactive drug, e.g. Table 2) [2] and uncertainty surrounding the composition of ingested drugs.

Past use of illicit drugs is likely to have contributed to overestimation of grip force in the stimulant group. The word ‘past’ is important here because subjects were not under the influence of a drug at the time of testing. Stimulants, opiates, and benzodiazepines were not present in urine samples obtained from subjects in the stimulant group and only 3 of the 22 subjects tested positive for cannabis (consumed 1 day prior to the experiment). Identification of the exact drug responsible is difficult in humans. In the current study, the most commonly used illicit stimulant drugs were methamphetamine (192±316 occasions) and ecstasy (75±121 occasions) but use of cannabis (2,246±2,770 occasions) and hallucinogens (60±154 occasions, mainly LSD) was also common. Furthermore, subjects in the stimulant group consumed more alcohol than subjects in the control groups. Increased consumption of alcohol is not unexpected as high levels of alcohol and tobacco use are well documented in this population [32]. However, the average rate of alcohol consumption in the stimulant group was well below the level that would induce brain damage. The dose of licit and illicit drugs is also likely to be important but only four subjects reported experiencing a drug overdose.

Of the drugs consumed, illicit stimulants are most likely to affect hand function due to their mechanism of action. There are a number of lines of evidence to support this view. Amphetamine, methamphetamine, and cocaine damage dopaminergic nerve terminals and chronic use of amphetamines is associated with long-lasting dopaminergic dysfunction [12], [33]. Animal and human studies also document changes in brain regions that control movement. Methamphetamine administration is associated with altered substantia nigra morphology in vervet monkeys [34] and adult humans with a history of illicit stimulant use also exhibit changes in this brain region [4]. Overestimation of grip force also occurs in patients newly diagnosed with Parkinson’s disease [16], a disease characterized by neurodegeneration of dopaminergic neurons in the substantia nigra [35]. History of illicit stimulant use is also associated with abnormal excitability in corticospinal projections to the hand of conscious humans [3] but the effect of illicit stimulant use on peripheral afferent input has not been investigated. These sorts of neurological changes may have functional consequences given that tremor during finger movement is abnormally large in individuals with a history of ecstasy use [5] and previous studies report impaired performance on the grooved pegboard test [36], [7], [6]. However, the latter finding was not replicated in the current study. Illicit stimulant drugs could act in isolation on hand function or they could interact with other licit and illicit drugs to alter performance.

In summary, our results suggest that individuals with a history of primarily methamphetamine and ecstasy use overestimate the grip force required to manipulate a novel object. The results provide additional evidence for an under recognized consequence of illicit stimulant use, movement dysfunction.
